# Antidiabetic and Anti-Inflammatory Effect of *Cinnamomum cassia* Oil in Alloxan-Induced Diabetic Rats

**DOI:** 10.3390/ph17091135

**Published:** 2024-08-29

**Authors:** Paula Cordero-Pérez, Flor Edith Hernández-Cruz, Daniel Garza-Guzmán, Diana Patricia Moreno-Peña, Concepción Sánchez-Martínez, Liliana Torres-González, Linda E. Muñoz-Espinosa, Homero Zapata-Chavira, Idalia Cura-Esquivel, Marisol Idalí Serrano-Sandoval, Diana Raquel Rodríguez-Rodríguez

**Affiliations:** 1 Liver Unit, Department of Internal Medicine, University Hospital “Dr. José E. González”, Universidad Autónoma de Nuevo León, Monterrey 64460, Nuevo León, Mexico; paucordero@yahoo.com.mx (P.C.-P.); daniel_garza95@yahoo.com (D.G.-G.); dpatricia.moreno@gmail.com (D.P.M.-P.); lilianator@gmail.com (L.T.-G.); linda_uanl@yahoo.com.mx (L.E.M.-E.);; 2 Nephrology Service, Department of Internal Medicine, University Hospital “Dr. José E. González”, Universidad Autónoma de Nuevo León, Monterrey 64460, Nuevo León, Mexico; dra_connie73@yahoo.com.mx; 3 Transplant Service, University Hospital “Dr. José E. González”, Universidad Autónoma de Nuevo León, Monterrey 64460, Nuevo León, Mexico; homero_zapata@yahoo.com; 4 Pediatric Service, University Hospital “Dr. José E. González”, Universidad Autónoma de Nuevo León, Monterrey 64460, Nuevo León, Mexico; idaliaaracely2008@hotmail.com

**Keywords:** *Cinnamomum cassia*, cinnamon, hypoglycemic, antidiabetic, hepatic, kidney, oxidative stress, anti-inflammatory

## Abstract

Diabetes mellitus presents a great diversity of treatments that cause adverse effects; therefore, plants are a source of compounds that may have fewer adverse effects; *Cinnamomum cassia* (*C. cassia*) has compounds with potential antidiabetic activity. The objective was to evaluate the antidiabetic effect of *C. cassia* oil (CCO) and its impact on oxidative stress in Wistar rats. Five groups were evaluated: (1) sham (SH), (2) 300 mg/kg CCO (CCO), (3) diabetic (D) induced with alloxan, (4) D + 300 mg/kg of CCO (D + CCO), and (5) D + 500 mg/kg of metformin (D + MET); all were treated for 5 days. CCO did not show alteration in aspartate aminotransferase (AST) and alanine aminotransferase (ALT) vs. SH. D + CCO vs. D significantly reduced glucose (333 ± 109 vs. 458 ± 81 mg/dL), ALT (66 ± 15 vs. 160 ± 54 U/L), AST (119 ± 26 vs. 243 ± 104 U/L), and blood urea nitrogen (18.8 ± 2.3 vs. 29.2 ± 6.9 mg/dL). No significant changes were observed in D + CCO vs. D in malondialdehyde (MDA), reduced glutathione (GSH), and superoxide dismutase (SOD), whereas a significant reduction in MDA and GSH was achieved in D + MET, with an increase in SOD. There was a reduction in *Rela* and *Gpx* in D + CCO and D + MET vs. D. CCO has antidiabetic and anti-inflammatory effects and reduces ALT, AST, and BUN levels.

## 1. Introduction

Diabetes mellitus (DM) is a disorder characterized by chronic hyperglycemia resulting from failure in insulin secretion, insulin action, or both, generating abnormalities in the metabolism of carbohydrates, fats, and proteins [1]. This hyperglycemia is related to acute (metabolic and infectious) or chronic complications, such as the dysfunction of various organs and tissues [2]. DM is a multifactorial disease, and in addition to genetic predisposition, there are environmental factors such as obesity, poor diet, and lack of exercise that contribute to the development of the disease [3].

In 2021, the International Diabetes Federation (IDF) reported that 537 million adults worldwide had diagnosed or undiagnosed DM. Mexico ranked seventh with the highest number of adults with DM, with 14.1 million cases. By 2045 worldwide, IDF projections indicate that approximately 783 million people will be living with diabetes (1 in 8 adults), which represents an increase of 46% [1]. In addition to its high incidence, type 2 DM (T2DM) has a strong economic impact, generating large expenses for public health systems [4,5].

In T2DM, reactive oxygen species (ROS) can accumulate and cause nonspecific oxidative damage to DNA, proteins, and other molecules, causing changes in their conformation and, therefore, changes in their functions [6,7]. Hyperglycemia also induces various mechanisms that lead to increased ROS production through processes such as glucose (GLU) autoxidation, protein glycation, and a decreased antioxidant defense system, such as superoxide dismutase (SOD), catalase, and glutathione peroxidase (GPx) [8], leading to an increase in free radicals, which is of utmost importance due to their relationship with the pathogenesis and appearance of complications of T2DM [7,9,10,11]. Gpx is a protein encoded by the *GPX* gene that belongs to the family of glutathione peroxidases, whose members catalyze the reduction of organic hydroperoxides and hydrogen peroxide by glutathione, thereby protecting cells against oxidative damage; it is known that in T2DM, this gene decreases its expression due to oxidative stress [12].

Chronic inflammation is another key factor in the progression of T2DM because, in tissues such as the liver, skeletal muscle, and adipose tissue, inflammation-dependent ROS interact with the insulin receptor and its signaling pathways, causing a failure of an adequate response to insulin levels [7,11]. Increased inflammatory biomarkers correlate with prevalent and incident T2DM and greater complications and cardiovascular diseases [13,14,15]. The increase in the nuclear factor kappa light chain enhancer of activated B cells (NFκβ), which has a fundamental role in the control and synthesis of proteins involved in the activation and maintenance of the inflammatory state, is due to the activation of intracellular signals of oxidative stress (OS) caused by increased protein kinase activity activated by the generation of reactive species [16]. NFκβ is composed of subunits, among which is the product of the *Rela* gene; the complex NFκβ remains inactive in the cytoplasm by binding of inhibitors. After the degradation of the inhibitor, this complex is translocated to the nucleus and activates the transcription of specific genes that promote inflammation [17].

There is currently no cure for T2DM; however, there are multiple treatments that aim to maintain normal GLU values to avoid or reduce the impact of related complications, although these have various adverse effects. For example, metformin can cause gastrointestinal effects and vitamin B12 deficiency, whereas sulfonylureas can cause hypoglycemia and weight gain [18,19]. In addition, it has been reported that these drugs can cause liver and kidney damage, and their use during pregnancy is not recommended. Recently, sodium–glucose cotransporter type 2 inhibitors have been a viable alternative with adequate therapeutic efficacy; however, their cost is very high for health systems [19]; hence, there is a need to search for new treatments where the compound’s natural sources may be a viable alternative [20,21,22,23,24].

Cinnamon has been one of the most studied plants for its hypoglycemic properties, being used as an adjuvant in the control of T2DM. It can improve GLU absorption by the activation of insulin receptor kinase, phosphorylation of this receptor, glycogen synthesis, and glycogen synthase activity in adipocytes in a Wistar rat model [25]. Among the most used cinnamon species are *Cinnamomum zeylanicum* (*C. zeylanicum*) or true cinnamon and *Cinnamomum cassia* (*C. cassia*) or Chinese cinnamon [26]. The latter has been reported to have antioxidant, antibacterial, anticancer, antifungal, antileishmanial, and antiviral activities, among others [27,28,29,30].

Oils are complex mixtures of secondary metabolites related to normal metabolic processes [31]; *C. cassia* oil (CCO) is mainly composed of *cis*-2-methoxycinnamic acid (43.06%), cinnamaldehyde (43.37%), *o*-methoxycinnamaldehyde (5.11%), 1,2-dimethoxy-4-(3-methoxy-1-propenyl)benzene (2.05%), and others such as eugenol [32,33,34]. Studies with cinnamon oil demonstrate improvements in fasting blood glucose, fasting insulin, and also the anatomy and function of kidney and liver cells; it has also been reported that cinnamaldehyde reduces GLU levels and increases insulin levels by increasing insulin-regulated glucose transporter protein type 4 in the fatty, liver, and muscle tissues of diabetic rats [31,35].

Other studies have determined that plants and fruits with hypoglycemic activity, such as maca, garlic, blueberries, and blackberries, produce an increase in antioxidant defense enzymes in protection against OS in animal models [36,37,38,39,40,41]. In the case of cinnamon, although several studies have evaluated its hypoglycemic effect, its effect on OS pathways and its modification in genetic expression have not been evaluated.

The aim of this study was to investigate the antidiabetic effect of CCO and its effect on OS pathways and its modification in gene expression. Diabetes was induced in Wistar rats using alloxan, which induces diabetes by damaging insulin-secreting pancreatic β cells, resulting in a decrease in endogenous insulin release and a decrease in GLU utilization by tissues, leading to hyperglycemia [42,43,44,45].

## 2. Results

**Figure 1:**
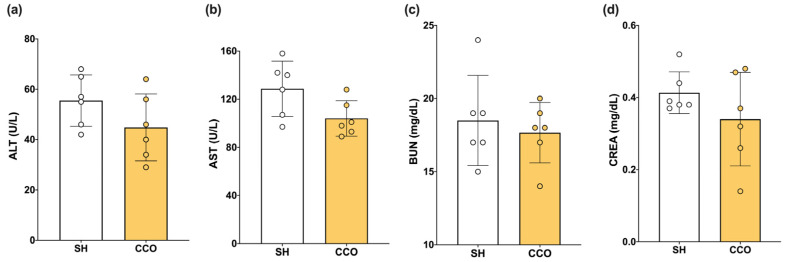
Effect of CCO administration on levels of ALT (**a**), AST (**b**), BUN (**c**), CREA (**d**). ALT: alanine aminotransferase, AST: aspartate aminotransferase, CREA: creatinine, BUN: blood urea nitrogen, SH: healthy control, and CCO: *C. cassia* oil. Values are expressed as mean ± SD.

To determine whether the dose of CCO used alone did not cause liver or kidney damage, biochemical parameters such as alanine aminotransferase (ALT), aspartate aminotransferase (AST), blood urea nitrogen (BUN), and creatinine (CREA) were measured. When evaluating the liver and kidney nontoxicity of CCO at the dose used, no significant difference in alanine aminotransferase (ALT), aspartate aminotransferase (AST), blood urea nitrogen (BUN), and creatinine (CREA) was observed between the sham (SH) and CCO in rats treated with 300 mg/kg of CCO for 5 days (Figure 1).

### 2.1. GLU Levels after 4 h of CCO Administration

**Figure 2:**
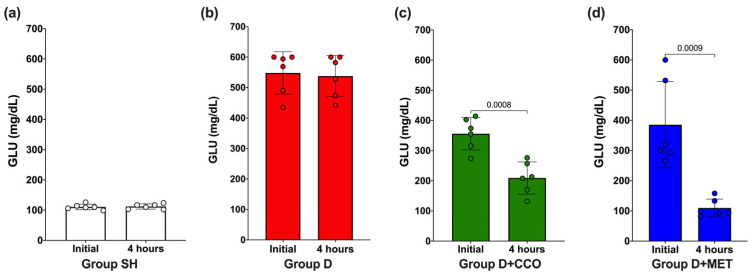
Effect on GLU levels after 4 h of CCO administration in SH group (**a**), D group (**b**), D + CCO group (**c**), D + MET group (**d**). GLU: glucose, SH: healthy control, D: diabetic, D + CCO: diabetic with *C. cassia* oil, and D + MET: diabetic with metformin. Values are expressed as mean ± SD.

To evaluate whether the dose of 300 mg/kg of CCO decreases GLU, an initial measurement was carried out and again at 4 h post-treatment. In SH and D, no significant difference in GLU was observed (Figure 2a and Figure 2b, respectively), whereas in D + CCO and D + MET, a significant decrease was observed at 4 h post-treatment (Figure 2c and Figure 2d, respectively).

### 2.2. CCO Decreases GLU Levels throughout the Treatment

**Figure 3:**
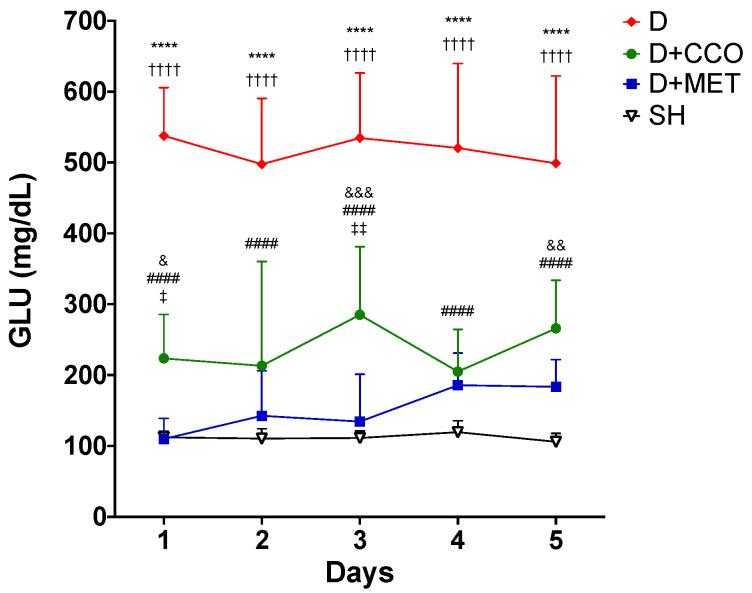
Daily levels of GLU in various study groups. GLU: glucose, SH: healthy control, D: diabetic, D + CCO: diabetic with *C. cassia* oil, and D + MET: diabetic with metformin. Values are expressed as mean ± SD. * SH vs. D; & SH vs. D + CCO; # D vs. D + CCO; † D vs. D + MET; ‡ D + CCO vs. D + MET. One symbol: *p* < 0.05; two symbols: *p* < 0.01; three symbols: *p* < 0.001; four symbols: *p* < 0.0001.

The administration of the CCO treatment was carried out for 5 days, to evaluate whether CCO decreases GLU levels in an acute model of diabetic rats, and it was observed that in D + CCO and D + MET, the decrease in daily GLU was maintained compared with D (Figure 3).

**Figure 4:**
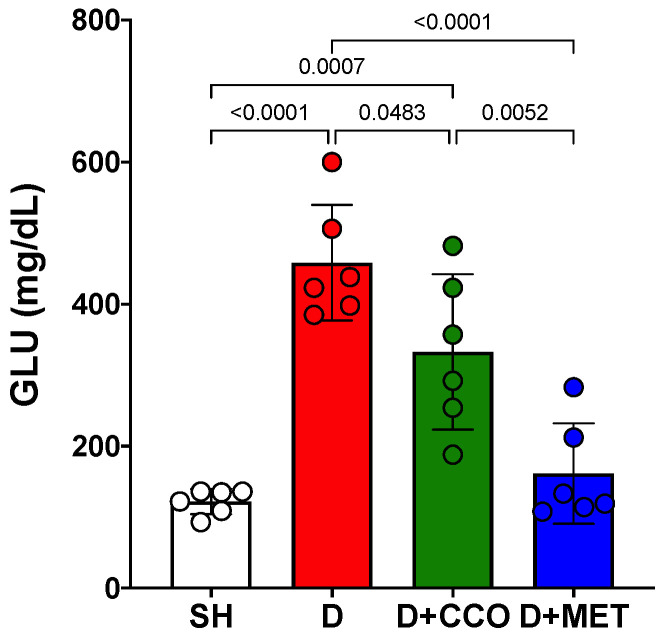
Final GLU levels in various study groups. GLU: glucose, SH: healthy control, D: diabetic, D + CCO: diabetic with *C. cassia* oil, and D + MET: diabetic with metformin. Values are expressed as mean ± SD.

Once the 5 days of treatment had elapsed, the final GLU measurement was carried out on the 6th day. A significant increase was observed in D vs. SH and a significant decrease in D + CCO and D + MET vs. D (Figure 4).

### 2.3. CCO on Liver and Kidney Function and Lipid Metabolism

**Figure 5:**
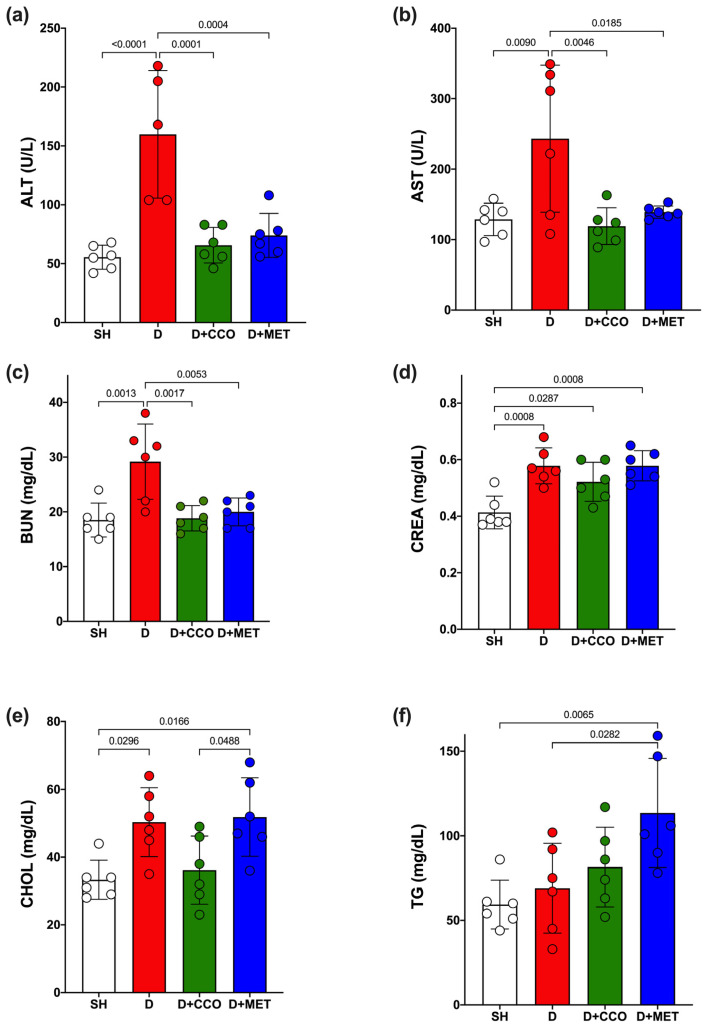
Serum levels of ALT (**a**), AST (**b**), BUN (**c**), CREA (**d**), CHOL (**e**) and TG (**f**) in study groups. ALT: alanine aminotransferase, AST: aspartate aminotransferase, BUN: blood urea nitrogen, CREA: creatinine, CHOL: cholesterol, TG: triglycerides, SH: healthy control, D: diabetic, D + CCO: diabetic with *C. cassia* oil, and D + MET: diabetic with metformin. Values are expressed as mean ± SD.

Some biochemical parameters were measured to determine how diabetes and CCO alter liver function, kidney function, and lipid metabolism. ALT and AST showed a significant increase in D vs. SH (Figure 5a,b). When the treatments were administered, both transaminases showed a significant decrease when comparing D + CCO and D + MET vs. D (Figure 5a,b). Furthermore, when comparing D + CCO against D + MET, no significant difference was observed for both enzymes, which means that both treatments work equally well for these.

Regarding BUN and CREA levels, a significant increase was observed in both parameters in D vs. SH (Figure 5c,d); however, a significant decrease in BUN was only observed in D + CCO and D + MET vs. D (Figure 5c). When comparing D + CCO vs. D + MET, no significant difference was observed for both BUN and CREA (Figure 5c,d). Therefore, treatment of diabetic rats with CCO and MET only improves BUN.

In the case of triglycerides (TG) and cholesterol (CHOL), only CHOL presented a significant increase in D vs. SH (Figure 5e); when comparing D + CCO and D + MET vs. D, only TG showed a significant increase in D + MET (Figure 5f). When comparing D + CCO vs. D + MET, only CHOL was significantly higher in D + MET (Figure 5e). As a result, CCO treatment in diabetic rats does not modify lipid metabolism.

### 2.4. Oxidative Stress Markers

**Figure 6:**
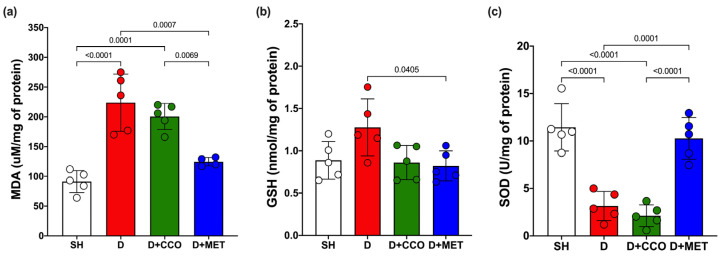
Levels of MDA (**a**), GSH (**b**), and SOD (**c**) in various study groups. MDA: malondialdehyde, GSH: reduced glutathione, SOD: superoxide dismutase, SH: healthy control, D: diabetic, D + CCO: diabetic with *C. cassia* oil, and D + MET: diabetic with metformin. Values are expressed as mean ± SD.

Malondialdehyde (MDA) is a biomarker to assess oxidative stress, along with the activities of GSH and SOD. MDA levels in liver tissue showed a significant increase in D vs. SH. Although a trend toward decreased MDA levels was observed in D + CCO and D + MET vs. D, only in D + MET was it significant, and when comparing D + CCO vs. D + MET, the MDA was also significantly lower in D + MET (Figure 6a).

Although GSH levels in D were higher than in SH, these were not statistically significant, and there was only a significant decrease in GSH in D + MET vs. D (Figure 6b).

In the measurement of SOD, there was a significant decrease in D vs. SH. When comparing D with D + CCO, there was no significant difference, whereas SOD in D + MET was significantly higher than in D and D + CCO, but without a significant difference with SH (Figure 6c). Therefore, CCO treatment in diabetic rats does not decrease MDA and GSH levels, and neither does it increase SOD levels.

### 2.5. Expression of Genes Associated with Inflammation and Oxidative Stress

**Figure 7:**
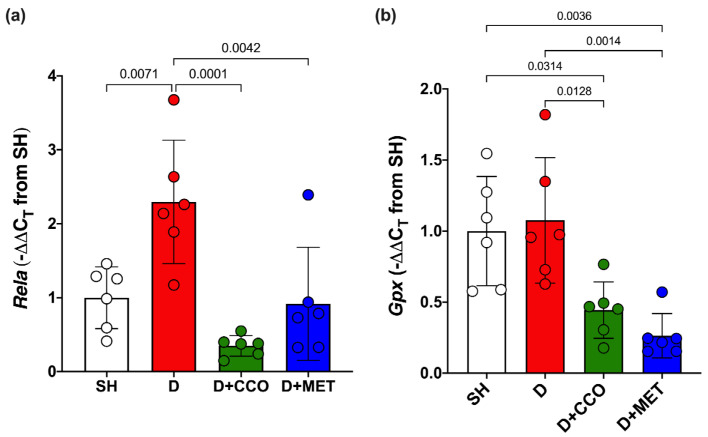
Expression of *Rela* (**a**) and *Gpx* (**b**) in different study groups. SH: healthy control, D: diabetic, D + CCO: diabetic with *C. cassia* oil, and D + MET: diabetic with metformin. Values are expressed as mean ± SD.

To evaluate inflammation and oxidative stress, *Rela* and *Gpx* gene expression were measured. In *Rela* gene expression, a significant increase was observed in D vs. SH, a significant decrease was observed in D + CCO and D + MET vs. D, and there was no significant difference between D + CCO and D + MET (Figure 7a). Regarding the gene expression of *Gpx* in D + CCO and D + MET, a significant decrease was observed vs. D, but not in D vs. SH. No significant difference was observed between D + CCO and D + MET (Figure 7b). Therefore, CCO treatment in diabetic rats decreases *Rela* and *Gpx* gene expression.

## 3. Discussion

Alloxan is used to elucidate the pathophysiology of diabetes and primarily serves as a tool for the antidiabetic compounds, and it is less expensive and easier to obtain than other diabetes inducers [46]. The model employs two distinct pathological mechanisms: a selective inhibition of glucose-stimulated insulin secretion through a specific inhibition of glucokinase, and an induced formation of reactive oxygen species (ROS) that promote selective necrosis of pancreatic beta cells; both effects are related to the chemical properties and structure of alloxan, collectively resulting in a pathophysiological state of diabetes [46,47,48].

Alloxan has been administered in single or multiple doses through various routes (intraperitoneal, intravenous, and subcutaneous), intraperitoneal administration being the most commonly used method. The drug dose varies between studies, ranging from 90 to 200 mg/kg, with 150 mg/kg being the most frequently used dose [46]. Doses above 160 have been associated with a high mortality rate and severe diabetes [43,46,49]. Our research group has experience with a dose of 120 mg/kg, so this dose was selected for this study [45]. 

It has been reported that in diabetic rat models induced with alloxan or streptozotocin (STZ), GLU levels must be greater than 225–250 mg/dL to be considered diabetic [44,45,50,51,52], which agrees with the results observed in the present study in the group treated with alloxan. This subsequently allowed us to evaluate the effect of CCO or metformin in diabetic rats.

It has been described that at high doses of the ethanolic extract of *C. cassia* bark (4000 and 5000 mg/kg), there is a slight increase in the concentration of ALT, AST, CREA, and urea; in addition, it was determined that a concentration of up to 3000 mg/kg does not present significant changes in liver or kidney parameters, so doses lower than this are considered safe [53]. At a dose of 300 mg/kg of CCO, there was no significant difference in the levels of AST, ALT, CREA, and BUN vs. SH, which agrees with previous reports on this plant.

In this study, diabetic rats administered CCO had their first glucose measurements taken at baseline and again at 4 h; this is because, in various models of alloxan-induced diabetes used to evaluate the antidiabetic activity of plant extracts, an antidiabetic effect has been reported within the first hours (1–8 h) after administration [54,55]. This effect has also been reported for various antidiabetic medications [44,45]. Additionally, several authors have shown that the administration of extracts containing antioxidant secondary metabolites can demonstrate an antidiabetic effect within 1 to 4 days post-administration [56,57]. Therefore, in this study, CCO was administered for 5 days to avoid prolonged stress in the experimental animal.

The aqueous extracts of *C. zeylanicum* and *C. cassia* have been shown to decrease GLU concentration in diabetic rat and db/db mouse models [58,59]. No studies were found that evaluated the effect of CCO on GLU concentration; however, it has been reported that *C. tamala* and *C. zeylanicum* oil have hypoglycemic properties [60,61]. In the present study, a significant decrease in GLU was observed in D + CCO compared with D, which coincides with the results previously reported with aqueous extracts of *C. cassia* and with oils from other *Cinnamomum* species. By contrast, D + MET presented a significant decrease in GLU compared with D, an expected result due to its hypoglycemic effect reported by other authors [50,51,62].

In DM, there is an increase in BUN due to greater protein catabolism, which has already been reported in diabetic rats induced with STZ and alloxan [52,63,64], coinciding with the significant increase in D vs. SH observed in the present study. By contrast, it has been reported that the aqueous extract of *C. cassia* in diabetic rats decreases BUN [65], and this was also observed in D + CCO.

CREA is the result of creatine metabolism and is used to evaluate kidney function. An increase in CREA has been reported in diabetic rats [52,63,66], which agrees with the present study where D presented a significant increase vs. SH. Methanolic and aqueous extracts of *Cinnamomum* decrease CREA levels in STZ-induced diabetic rats treated for 28 days [67,68], which differs from the present study where there was no decrease in this parameter, perhaps due to the 5-day treatment time.

T2DM produces an increase in CHOL and TG. A significant elevation of CHOL in short study periods (less than one month) has been reported in diabetic Wistar rats, whereas TG increased over longer periods of time (greater than two months) [69]. This agrees with the present study where an increase in CHOL but not TG was observed, attributed to the short treatment time. Other studies have reported a decrease in CHOL and TG after the administration of *C. cassia* for 4–8 weeks [68,70]; however, in the present study, no significant decrease in CHOL was observed.

In liver damage, there is an increase in AST and ALT associated with insulin resistance, metabolic syndrome, and diabetes [71]. In this study, alloxan, which is metabolized by the liver, was used as a diabetes inducer. The liver presents various strategies for protection against ROS; it also biotransforms and eliminates xenobiotics; therefore, the increase in transaminases is attributed to DM [72]. In this study, a significant increase in transaminases in D was observed, attributed to the damage caused by DM and not by alloxan, as previously described [71,72]. Cinnamon has been shown to reduce ALT and AST levels in diabetic rat models [53,58], which is consistent with the present study.

In T2DM, there is an increase in various reactive species, which when accumulated cause oxidative damage, such as lipid peroxidation. One of the most used markers to evaluate this damage is MDA [7], and its increase has been reported in diabetic rats [73,74,75], which is observed in the present study. It has been reported that *C. cassia* decreases MDA in tissues such as the kidney, liver, and thoracic aorta of diabetic animals [20,74,75]; however, this differs from the results obtained in the present study. Despite observing a tendency to decrease MDA in D + CCO, this was not significant, but it was significant for D + MET. This discrepancy could be because in the previously cited studies, the administration periods were between 35 and 84 days, in contrast to the present study where it was only administered for 5 days.

The decrease in the antioxidant defense system is another mechanism of T2DM related to the increase in free radicals, involved in the pathogenesis of this disease [6]. SOD is an important antioxidant defense in most cells exposed to oxygen; a decrease in SOD levels has been reported in both the kidney and liver of diabetic rats [58,74], which agrees with the results observed for this enzyme in D. It has been reported that *C. cassia* at doses of 100–400 mg/kg for 6–12 weeks increases SOD in the liver and kidney of diabetic rats [20,58,74]; this differed from the results obtained in D + CCO, which may be because the previously reported studies used aqueous and methanolic extracts and not CCO, in addition to the short administration period evaluated in the present study.

By contrast, GSH, a cofactor necessary for Gpx activity, is decreased in diabetic rats in study periods of 5–6 weeks [74,75], which differs from the results obtained in the present study, in which an increase in GSH was observed but without a significant difference in D vs. SH; this could be attributed to the short follow-up period evaluated in our study (5 days). *C. cassia* has been shown to increase GSH levels [20,74,75]. In our study, it was observed that in D + CCO and D + MET, GSH levels were similar to SH, and there was only a significant difference between D and D + MET. This is possibly due to the conditions already referred to with MDA and SOD. In addition to the study period, another aspect that could influence the results related to OS is the composition of the CCO. Although cinnamon has antioxidants such as proanthocyanidin A and flavonoids [76], these molecules are very polar, so they would not be present in the CCO due to their nonpolar nature.

Regarding the expression of genes associated with inflammation and OS in T2DM, in the case of *Rela*, an increase has been reported in diabetic rats [77,78], consistent with the results observed in D, which presented a significant increase vs. SH. The effect of *C. cassia* on the relative expression of *Rela* has not been described. However, it has been reported that the methanolic extract of *C. cassia* has an inhibitory effect on the protein expression of *Rela* in an in vitro model with RAW 264.7 macrophages, which correlates with a lower expression of the gene [79]. This coincides with the results obtained in vivo, in which a significant decrease in *Rela* expression was observed in D + CCO vs. D.

By contrast, a decrease in *Gpx* expression has been reported in diabetic rat models treated for 8 weeks [80]. In the present study, there was no significant difference between SH and D, possibly due to the short study period. No data were found about the effect of CCO or any *C. cassia* extract on the activity of this gene. However, it has been reported that *C. zeylanicum* powder administered to Japanese quail for 84 days [81], and an aqueous suspension of *C. zeylanicum* powder administered to diabetic rats for 2 weeks, increases the expression of *Gpx* [82], which differs from this study, where there was a significant decrease in the expression of *Gpx* in D + CCO vs. D. This could be due to the time of administration or the composition of the oil vs. powder.

CCO significantly decreased glucose, BUN, AST, and ALT levels, and *Rela* gene expression in diabetic rats. This antidiabetic effect could be attributable to the stimulation of the remaining β cells or possibly to an enhanced cellular utilization of glucose, as reported by other authors [45,83]. This effect could be due to cinnamaldehyde, one of the major compounds present in CCO, which has already been reported to have hypoglycemic properties [31,35]. MET was better than CCO in controlling GLU, MDA, and SOD levels, but not in lipid metabolism, where TG levels were significantly increased compared to group D. One perspective of this work is the evaluation of CCO in a chronic diabetes model to establish the possible signaling pathways involved in the activity of *C. cassia.*

## 4. Materials and Methods

### 4.1. Chemicals, Reagents, and Sample Size

Metformin (Merck S.A. de C.V., Mexico, Mexico), glucose (CTR Scientific, Monterrey, N.L., Mexico), CCO, and alloxan monohydrate (Sigma-Aldrich, Saint Louis, MO, USA) were purchased commercially.

Thirty Wistar rats, weighing between 180 and 300 g, were used in this study. They were divided into five mixed-gender groups (three females and three males) to ensure that the observed effect was not influenced by physiological gender differences, hormonal variations between sexes, or the fact that female rats may have an intrinsically greater antioxidant capacity [84,85,86]. The animals were housed in polyethylene cages at a temperature of 24 ± 3 °C with 12 h light and dark cycles. The rats were fed a commercial diet in pellets (Nutrimix de Mexico, S.A. de C.V., Mexico City, Mexico) and water ad libitum until they were classified into the different groups to be used. The experiments were carried out in accordance with NOM-062-ZOO-1999 and approved by the Ethics and Research Committee of the Faculty of Medicine of the Autonomous University of Nuevo León with registration number HI17-00002.

### 4.2. Diabetes Induction

For the induction of diabetes, food was withdrawn from the rats 4 h before the administration of 120 mg/kg of alloxan monohydrate dissolved in 1 mL of a 0.9% saline solution (SS) via the intraperitoneal (ip) route; GLU was monitored for 7 days. A cutoff value of >250 mg/dL of GLU was considered to classify the rats as diabetic. The alloxan dose used and the glucose levels to classify the rats as diabetic were selected based on previous reports by other authors [44,45,50,51,52].

### 4.3. Experimental Groups

(1) Sham (SH) group: 1 mL of 0.9% SS was administered ip, in addition to 1 mL of 0.9% SS intragastrically for 5 days. Free access to pellets and tap water throughout the study period.

(2) *C. cassia* oil (CCO) group: 1 mL of 0.9% SS was administered ip; in addition, 300 mg/kg of CCO in 1 mL of 0.9% SS was administered intragastrically for 5 days. Free access to pellets and tap water throughout the study period.

(3) Diabetic (D) group: 120 mg/kg of alloxan dissolved in 1 mL of 0.9% SS was administered ip, in addition to 1 mL of 0.9% SS intragastrically for 5 days. Free access to pellets and water with 5% d-glucose throughout the study period.

(4) Diabetic group treated with metformin (D + MET): 120 mg/kg of alloxan dissolved in 1 mL of 0.9% SS was administered ip, and 1 mL of 0.9% SS intragastrically for 5 days. In addition, 500 mg/kg of metformin dissolved in 1 mL of 0.9% SS was administered intragastrically for 5 days. Free access to pellets and water with 5% d-glucose throughout the study period.

(5) Diabetic group treated with *C. cassia* oil (D + CCO): 120 mg/kg of alloxan dissolved in 1 mL of 0.9% SS was administered ip. In addition, 300 mg/kg of CCO dissolved in 1 mL of 0.9% SS was administered intragastrically for 5 days. Free access to pellets and water with 5% d-glucose throughout the study period. The CCO dose was chosen based on previous reports by other authors [53,68].

Once the study period was completed, the animals were anesthetized with 10 mg/kg xylazine and 100 mg/kg ketamine ip. Subsequently, anesthesia was checked by clamping the hind leg and/or pinching the tail. Once anesthesia was confirmed, the animals were taken to the surgical table where the abdomen was shaved and the skin was cleaned with Microdacyn 60 and chlorhexidine. Then, a laparotomy was performed by making an incision in the abdomen; the vena cava was located, from which blood was collected by puncture (exsanguination), obtaining the largest volume of blood possible, thus leading to euthanasia. The serum was kept frozen at –80 °C until the analysis. In addition, the liver was removed and immediately frozen at –80 °C until the analysis.

GLU, CREA, BUN, TG, CHOL, AST, and ALT were determined from serum. In liver tissue, the measurement of the OS markers MDA, GSH, and SOD as well as the determination of the gene expression of *Rela* and *Gpx* were carried out.

### 4.4. GLU Measurement

Blood GLU was measured by puncturing the tip of the rats’ tails, extracting a drop of blood, and placing it on the test strip of the Accu-Check Performa digital glucometer (Roche Diagnostics, SL, Barcelona, Spain).

GLU measurements were carried out on day–7 (baseline), after one week of the administration of alloxan (group D) or SS (groups SH and CCO), until reaching a concentration >250 mg/dL in D. Subsequently, for the groups that met the inclusion criterion, the administration of the treatment (groups D + MET and D + CCO) or vehicle (groups SH and D) began. In addition, GLU measurements were performed 4 h after the treatments (day 1) and on subsequent days (days 2, 3, 4, and 5) 1 h after administration.

### 4.5. Determination of Biochemical Markers

Serum samples from all study groups were analyzed on the Ilab Aries equipment (Instrumentation Laboratory, Werfen Group, Barcelona, Spain) for the determination of GLU, TG, CHOL, AST, ALT, BUN, and CREA using UV–visible spectrophotometry.

### 4.6. Determination of Oxidative Stress Markers

The determination of MDA was performed in the tissue homogenate using the trichloroacetic acid method, which is based on the reaction of MDA with thiobarbituric acid, with the TBARS assay kit (Cayman Chemical Company, Ann Arbor, MI, USA). The product of this reaction was measured spectrophotometrically at a wavelength of 535 nm in a Multiskan GO (Thermo Fisher Scientific, Carlsbad, CA, USA), its concentration being proportional to the concentration of MDA in the sample.

SOD activity was determined by the tetrazolium salt reduction method to formazan using a superoxide dismutase assay kit (Sigma-Aldrich). SOD activity was quantified as inhibitory activity by measuring formazan depletion using spectrophotometry at a wavelength of 440 nm on a Multiskan GO.

For the measurement of GSH, a reduced glutathione assay kit (Sigma-Aldrich) was used through a kinetic assay, in which GSH causes the continuous reduction of 5,5′-dithio-bis-[2-nitrobenzoic acid] to 2-nitro-5-thiobenzoic acid (TNB), in addition to forming glutathione disulfide as a product of the oxidation of GSH, which is reduced by glutathione reductase in the presence of Nicotinamide-Adenine Dinucleotide Phosphate. The TNB formed was quantified by spectrophotometry at 412 nm on a Multiskan GO.

All measurements in tissue were normalized to the protein concentration in the homogenates, measured using Bradford’s method. Bradford’s reagent was prepared as previously reported in the literature [87].

### 4.7. RNA Extraction and qRT-PCR

RNA extraction was performed from 100 mg of frozen tissue using the Trizol reagent (Invitrogen, Thermo Fisher Scientific, Carlsbad, CA, USA) according to the manufacturer’s instructions. RNA was precipitated with 100% 2-propanol, washed with 75% ethanol, resuspended in RNase-free water, and quantified in a Microdrop Multiskan GO (Thermo Fisher Scientific). For qRT-PCR, 100 ng of RNA, a commercial one-step qRT-PCR kit (Promega Corporation, Madison, WI, USA), and 50–200 nM primers were used to measure the gene expression of *Rela*, *Gpx*, and *Actb*. The forward *Rela* primer used was 5′-CCTCATCTTTCCCTCAGAGCC-3′, reverse *Rela*: 5′-CGCACTTGTAACGGAAACGC-3′, forward *Gpx*: 5′-CATTGAGAATGTCGCGTCCC-3′, reverse *Gpx*: 5′-TTGCCATTCTCCTGATGTCCG-3′, forward *Actb*: 5′-CCCTGGCTCCTAGCACCAT-3′, and reverse *Actb*: 5′-GATAGAGCCACCAATCCACACA-3′ [88].

### 4.8. Statistical Analysis

The data were analyzed using GraphPad Prism 7.0 Software. For all study groups, a Shapiro–Wilk normality test, a *T* test for the comparison of two groups, and a one-way analysis of variance with a Tukey post hoc test were performed for comparisons between three or more groups. The results are expressed as the mean ± standard deviation, considering a value of *p* < 0.05 as statistically significant.

## 5. Conclusions

In summary, it was determined that CCO at a dose of 300 mg/kg does not cause liver or kidney damage; and in diabetic rats, it reduced ALT, AST, and BUN levels, and had antidiabetic and anti-inflammatory effects. Our results suggest the potential of CCO to be developed as a natural antidiabetic drug; however, further long-term animal research, as well as preclinical and clinical trials, is required to clearly demonstrate the activity of CCO.

## Data Availability

The original contributions presented in the study are included in the article; further inquiries can be directed to the corresponding author.

## References

[1] Boyko E.J., Magliano D.J., Karuranga S., Piemonte L., Pouya Saeedi P.R., Sun H., International Diabetes Federation (2021). IDF Diabetes Atlas.

[2] López Stewart G. (2009). Diabetes Mellitus: Clasificación, Fisiopatología y Diagnóstico. Medwave.

[3] Leong A., Porneala B., Dupuis J., Florez J.C., Meigs J.B. (2016). Type 2 Diabetes Genetic Predisposition, Obesity, and All-Cause Mortality Risk in the U.S.: A Multiethnic Analysis. Diabetes Care.

[4] Mendoza Romo M.Á., Padrón Salas A., Cossío Torres P.E., Soria Orozco M. (2018). Prevalencia Mundial de La Diabetes Mellitus Tipo 2 y Su Relación Con El Índice de Desarrollo Humano. Rev. Panam. Salud Pública.

[5] Williams R., Karuranga S., Malanda B., Saeedi P., Basit A., Besançon S., Bommer C., Esteghamati A., Ogurtsova K., Zhang P. (2020). Global and Regional Estimates and Projections of Diabetes-Related Health Expenditure: Results from the International Diabetes Federation Diabetes Atlas, 9th Edition. Diabetes Res. Clin. Pr..

[6] Darenskaya M.A., Kolesnikova L.I., Kolesnikov S.I. (2021). Oxidative Stress: Pathogenetic Role in Diabetes Mellitus and Its Complications and Therapeutic Approaches to Correction. Bull. Exp. Biol. Med..

[7] Newsholme P., Cruzat V.F., Keane K.N., Carlessi R., de Bittencourt P.I.H. (2016). Molecular Mechanisms of ROS Production and Oxidative Stress in Diabetes. Biochem. J..

[8] Khalili F., Vaisi-Raygani A., Shakiba E., Kohsari M., Dehbani M., Naseri R., Asadi S., Rahimi Z., Rahimi M., Rahimi Z. (2022). Oxidative Stress Parameters and Keap 1 Variants in T2DM: Association with T2DM, Diabetic Neuropathy, Diabetic Retinopathy, and Obesity. J. Clin. Lab. Anal..

[9] Singh A., Kukreti R., Saso L., Kukreti S. (2022). Mechanistic Insight into Oxidative Stress-Triggered Signaling Pathways and Type 2 Diabetes. Molecules.

[10] Luc K., Schramm-Luc A., Guzik T.J., Mikolajczyk T.P. (2019). Oxidative Stress and Inflammatory Markers in Prediabetes and Diabetes. J. Physiol. Pharmacol..

[11] Newsholme P., Cruzat V., Arfuso F., Keane K. (2014). Nutrient Regulation of Insulin Secretion and Action. J. Endocrinol..

[12] Sadi G., Güray T. (2009). Gene Expressions of Mn-SOD and GPx-1 in Streptozotocin-Induced Diabetes: Effect of Antioxidants. Mol. Cell Biochem..

[13] Lontchi-Yimagou E., Sobngwi E., Matsha T.E., Kengne A.P. (2013). Diabetes Mellitus and Inflammation. Curr. Diabetes Rep..

[14] Karam B.S., Chavez-Moreno A., Koh W., Akar J.G., Akar F.G. (2017). Oxidative Stress and Inflammation as Central Mediators of Atrial Fibrillation in Obesity and Diabetes. Cardiovasc. Diabetol..

[15] Guo W., Song Y., Sun Y., Du H., Cai Y., You Q., Fu H., Shao L. (2022). Systemic Immune-Inflammation Index Is Associated with Diabetic Kidney Disease in Type 2 Diabetes Mellitus Patients: Evidence from NHANES 2011–2018. Front. Endocrinol..

[16] Lingappan K. (2018). NF-ΚB in Oxidative Stress. Curr. Opin. Toxicol..

[17] Sun Z., Andersson R. (2002). NF-KappaB Activation and Inhibition: A Review. Shock.

[18] Rosenstock J., Allison D., Birkenfeld A.L., Blicher T.M., Deenadayalan S., Jacobsen J.B., Serusclat P., Violante R., Watada H., Davies M. (2019). Effect of Additional Oral Semaglutide vs Sitagliptin on Glycated Hemoglobin in Adults with Type 2 Diabetes Uncontrolled with Metformin Alone or with Sulfonylurea: The PIONEER 3 Randomized Clinical Trial. JAMA.

[19] American Diabetes Association Professional Practice Committee 9 (2024). Pharmacologic Approaches to Glycemic Treatment: Standards of Care in Diabetes-2024. Diabetes Care.

[20] Kim S.H., Hyun S.H., Choung S.Y. (2006). Antioxidative Effects of Cinnamomi Cassiae and Rhodiola Rosea Extracts in Liver of Diabetic Mice. Biofactors.

[21] Xu L., Li Y., Dai Y., Peng J. (2018). Natural Products for the Treatment of Type 2 Diabetes Mellitus: Pharmacology and Mechanisms. Pharmacol. Res..

[22] Ríos J.L., Francini F., Schinella G.R. (2015). Natural Products for the Treatment of Type 2 Diabetes Mellitus. Planta Med..

[23] Watanabe S., Okoshi H., Yamabe S., Shimada M. (2021). *Moringa oleifera* Lam. in Diabetes Mellitus: A Systematic Review and Meta-Analysis. Molecules.

[24] Patle D., Vyas M., Khatik G.L. (2021). A Review on Natural Products and Herbs Used in the Management of Diabetes. Curr. Diabetes Rev..

[25] Qin B., Nagasaki M., Ren M., Bajotto G., Oshida Y., Sato Y. (2003). Cinnamon Extract (Traditional Herb) Potentiates In Vivo Insulin-Regulated Glucose Utilization via Enhancing Insulin Signaling in Rats. Diabetes Res. Clin. Pr..

[26] Balijepalli M.K., Buru A.S., Sakirolla R., Pichika M.R. (2017). Cinnamomum Genus: A Review on Its Biological Activities. Int. J. Pharm. Pharm. Sci..

[27] Alam A., Ansari M.J., Alqarni M.H., Salkini M.A., Raish M. (2023). Antioxidant, Antibacterial, and Anticancer Activity of Ultrasonic Nanoemulsion of *Cinnamomum cassia* L. Essential Oil. Plants.

[28] Minozzo M., de Souza M.A., Bernardi J.L., Puton B.M.S., Valduga E., Steffens C., Paroul N., Cansian R.L. (2023). Antifungal Activity and Aroma Persistence of Free and Encapsulated *Cinnamomum cassia* Essential Oil in Maize. Int. J. Food Microbiol..

[29] Afrin F., Chouhan G., Islamuddin M., Want M.Y., Ozbak H.A., Hemeg H.A. (2019). *Cinnamomum cassia* Exhibits Antileishmanial Activity against Leishmania Donovani Infection In Vitro and In Vivo. PLoS Negl. Trop. Dis..

[30] Fatima M., Zaidi N.-U.-S.S., Amraiz D., Afzal F. (2016). In Vitro Antiviral Activity of *Cinnamomum cassia* and Its Nanoparticles Against H7N3 Influenza A Virus. J. Microbiol. Biotechnol..

[31] Stevens N., Allred K. (2022). Antidiabetic Potential of Volatile Cinnamon Oil: A Review and Exploration of Mechanisms Using In Silico Molecular Docking Simulations. Molecules.

[32] Chang C.-T., Chang W.-L., Hsu J.-C., Shih Y., Chou S.-T. (2013). Chemical Composition and Tyrosinase Inhibitory Activity of *Cinnamomum cassia* Essential Oil. Bot. Stud..

[33] Verspohl E.J., Bauer K., Neddermann E. (2005). Antidiabetic Effect of *Cinnamomum cassia* and *Cinnamomum zeylanicum* In Vivo and In Vitro. Phytotherapy Res..

[34] Yan Y.-M., Fang P., Yang M.-T., Li N., Lu Q., Cheng Y.-X. (2015). Anti-Diabetic Nephropathy Compounds from *Cinnamomum cassia*. J. Ethnopharmacol..

[35] Subash Babu P., Prabuseenivasan S., Ignacimuthu S. (2007). Cinnamaldehyde—A Potential Antidiabetic Agent. Phytomedicine.

[36] Mohamed S.M., Shalaby M.A., El-Shiekh R.A., Bakr A.F., Kamel S., Emam S.R., El-Banna H.A. (2024). Maca Roots: A Potential Therapeutic in the Management of Metabolic Disorders through the Modulation of Metabolic Biochemical Markers in Rats Fed High-Fat High-Carbohydrate Diet. J. Ethnopharmacol..

[37] Drobiova H., Thomson M., Al-Qattan K., Peltonen-Shalaby R., Al-Amin Z., Ali M. (2011). Garlic Increases Antioxidant Levels in Diabetic and Hypertensive Rats Determined by a Modified Peroxidase Method. Evid.-Based Complement. Altern. Med..

[38] Thomson M., Al-Qattan K.K., Js D., Ali M. (2016). Anti-Diabetic and Anti-Oxidant Potential of Aged Garlic Extract (AGE) in Streptozotocin-Induced Diabetic Rats. BMC Complement. Altern. Med..

[39] Rahmani G., Farajdokht F., Mohaddes G., Babri S., Ebrahimi V., Ebrahimi H. (2020). Garlic (*Allium sativum*) Improves Anxiety- and Depressive-Related Behaviors and Brain Oxidative Stress in Diabetic Rats. Arch. Physiol. Biochem..

[40] Mirazi N., Hosseini A. (2020). Attenuating Properties of *Rubus fruticosus* L. on Oxidative Damage and Inflammatory Response Following Streptozotocin-Induced Diabetes in the Male Wistar Rats. J. Diabetes Metab. Disord..

[41] Chehri A., Yarani R., Yousefi Z., Shakouri S.K., Ostadrahimi A., Mobasseri M., Araj-Khodaei M. (2022). Phytochemical and Pharmacological Anti-Diabetic Properties of Bilberries (*Vaccinium myrtillus*), Recommendations for Future Studies. Prim. Care Diabetes.

[42] Chatzigeorgiou A., Halapas A., Kalafatakis K., Kamper E. (2009). The Use of Animal Models in the Study of Diabetes Mellitus. In Vivo.

[43] Szkudelski T. (2001). The Mechanism of Alloxan and Streptozotocin Action in B Cells of the Rat Pancreas. Physiol. Res..

[44] Trevino-Moreno S.G., Moreno-Peña D.P., Viveros-Valdez E., Verde-Star M.J., Rivas-Morales C., Cordero-Perez P. (2023). Evaluation of Hypoglycemic and Antioxidant Effects of *Brickellia eupatorioides*, *Citrus limettioides* and *Gochnatia hypoleuca*. Pak. J. Pharm. Sci..

[45] Rodríguez-Magaña M.P., Cordero-Pérez P., Rivas-Morales C., Oranday-Cárdenas M.A., Moreno-Peña D.P., García-Hernández D.G., Leos-Rivas C. (2019). Hypoglycemic Activity of *Tilia americana*, *Borago officinalis*, *Chenopodium nuttalliae*, and *Piper sanctum* on Wistar Rats. J. Diabetes Res..

[46] Ighodaro O.M., Adeosun A.M., Akinloye O.A. (2017). Alloxan-Induced Diabetes, a Common Model for Evaluating the Glycemic-Control Potential of Therapeutic Compounds and Plants Extracts in Experimental Studies. Medicina.

[47] Jörns A., Munday R., Tiedge M., Lenzen S. (1997). Comparative Toxicity of Alloxan, N-Alkylalloxans and Ninhydrin to Isolated Pancreatic Islets In Vitro. J. Endocrinol..

[48] Shaw Dunn J., Mcletchie N.G.B. (1943). Experimental Alloxan Diabetes in the Rat. Lancet.

[49] Chougale A.D., Panaskar S.N., Gurao P.M., Arvindek A.U. (2007). Optimization of Alloxan Dose Is Essential to Induce Stable Diabetes for Prolonged Period. Asian J. Biochem..

[50] He X., Gao F., Hou J., Li T., Tan J., Wang C., Liu X., Wang M., Liu H., Chen Y. (2021). Metformin Inhibits MAPK Signaling and Rescues Pancreatic Aquaporin 7 Expression to Induce Insulin Secretion in Type 2 Diabetes Mellitus. J. Biol. Chem..

[51] Ahmed Mobasher M., Galal El-Tantawi H., Samy El-Said K. (2020). Metformin Ameliorates Oxidative Stress Induced by Diabetes Mellitus and Hepatocellular Carcinoma in Rats. Rep. Biochem. Mol. Biol..

[52] Tan H., Chen J., Li Y., Li Y., Zhong Y., Li G., Liu L., Li Y. (2022). Glabridin, a Bioactive Component of Licorice, Ameliorates Diabetic Nephropathy by Regulating Ferroptosis and the VEGF/Akt/ERK Pathways. Mol. Med..

[53] Vijayakumar K., Rengarajan R.L., Suganthi N., Prasanna B., Velayuthaprabhu S., Shenbagam M., Vijaya Anand A. (2022). Acute Toxicity Studies and Protective Effects of *Cinnamon cassia* Bark Extract in Streptozotocin-Induced Diabetic Rats. Drug Chem. Toxicol..

[54] Castañeda B.C., de la Mata R.C., Mejia R.M., Vasquez L.I., Alarcón R.F., Mendoza E.B. (2008). Estudio Fitoquímico y Farmacológico de 4 Plantas Con Efecto Hipoglicemiante. Horiz. Médico.

[55] Rao B.K., Kesavulu M.M., Apparao C. (2001). Antihyperglycemic Activity of Momordica Cymbalaria in Alloxan Diabetic Rats. J. Ethnopharmacol..

[56] Jawla S., Kumar Y., Khan M.S. (2011). Antimicrobial and Antihyperglycemic Activities of Acacia Modesta Leaves. Pharmacologyonline.

[57] Kingsley R.B., Jesuraj SA V., Brindha P., Subramoniam A., Atif M. (2013). Anti-Diabetes Activity of *Acacia farnesiana* (L.) Willd in Alloxan Diabetic Rats. Int. J. PharmTech Res..

[58] Shalaby M., Saifan H. (2014). Some Pharmacological Effects of Cinnamon and Ginger Herbs in Obese Diabetic Rats. J. Intercult. Ethnopharmacol..

[59] Kim S.H., Hyun S.H., Choung S.Y. (2006). Anti-Diabetic Effect of Cinnamon Extract on Blood Glucose in Db/Db Mice. J. Ethnopharmacol..

[60] Kumar S., Vasudeva N., Sharma S. (2012). GC-MS Analysis and Screening of Antidiabetic, Antioxidant and Hypolipidemic Potential of *Cinnamomum tamala* Oil in Streptozotocin Induced Diabetes Mellitus in Rats. Cardiovasc. Diabetol..

[61] Mohammed K.A.A., Ahmed H.M.S., Sharaf H.A., El-Nekeety A.A., Abdel-Aziem S.H., Mehaya F.M., Abdel-Wahhab M.A. (2020). Encapsulation of Cinnamon Oil in Whey Protein Counteracts the Disturbances in Biochemical Parameters, Gene Expression, and Histological Picture of the Liver and Pancreas of Diabetic Rats. Environ. Sci. Pollut. Res..

[62] Ren H., Shao Y., Wu C., Ma X., Lv C., Wang Q. (2020). Metformin Alleviates Oxidative Stress and Enhances Autophagy in Diabetic Kidney Disease via AMPK/SIRT1-FoxO1 Pathway. Mol. Cell. Endocrinol..

[63] Sachan R., Kundu A., Dey P., Son J.Y., Kim K.S., Lee D.E., Kim H.R., Park J.H., Lee S.H., Kim J.H. (2020). *Dendropanax morbifera* Protects against Renal Fibrosis in Streptozotocin-Induced Diabetic Rats. Antioxidants.

[64] Sajid M., Khan M.R., Ismail H., Latif S., Rahim A.A., Mehboob R., Shah S.A. (2020). Antidiabetic and Antioxidant Potential of *Alnus nitida* Leaves in Alloxan Induced Diabetic Rats. J. Ethnopharmacol..

[65] Nagai H., Shimazawa T., Takizawa T., Koda A., Yagi A., Nishioka I. (1982). Immunopharmacological Studies of the Aqueous Extract of *Cinnamomum cassia* (CCAq). II. Effect of CCAq on Experimental Glomerulonephritis. Jpn. J. Pharmacol..

[66] Ganesan D., Albert A., Paul E., Ananthapadmanabhan K., Andiappan R., Sadasivam S.G. (2020). Rutin Ameliorates Metabolic Acidosis and Fibrosis in Alloxan Induced Diabetic Nephropathy and Cardiomyopathy in Experimental Rats. Mol. Cell Biochem..

[67] Qusti S., El Rabey H.A., Balashram S.A. (2016). The Hypoglycemic and Antioxidant Activity of Cress Seed and Cinnamon on Streptozotocin Induced Diabetes in Male Rats. Evid.-Based Complement. Altern. Med..

[68] Vijayakumar K., Prasanna B., Rengarajan R.L., Rathinam A., Velayuthaprabhu S., Vijaya Anand A. (2023). Anti-Diabetic and Hypolipidemic Effects of *Cinnamon cassia* Bark Extracts: An In Vitro, In Vivo, and In Silico Approach. Arch. Physiol. Biochem..

[69] Figueroa M.C., Pérez I.H., Mejía R. (2013). Caracterización de Un Modelo de Diabetes Tipo 2 En Ratas Wistar Hembra. Rev. MVZ Córdoba.

[70] Shatwan I.A., Ahmed L.A., Badkook M.M. (2013). Effect of Barley Flour, Crude Cinnamon, and Their Combination on Glycemia, Dyslipidemia, and Adipose Tissue Hormones in Type 2 Diabetic Rats. J. Med. Food.

[71] Vozarova B., Stefan N., Lindsay R.S., Saremi A., Pratley R.E., Bogardus C., Tataranni P.A. (2002). High Alanine Aminotransferase Is Associated with Decreased Hepatic Insulin Sensitivity and Predicts the Development of Type 2 Diabetes. Diabetes.

[72] Kottaisamy C.P.D., Raj D.S., Prasanth Kumar V., Sankaran U. (2021). Experimental Animal Models for Diabetes and Its Related Complications—A Review. Lab. Anim. Res..

[73] Arkali G., Aksakal M., Kaya Ş.Ö. (2021). Protective Effects of Carvacrol against Diabetes-Induced Reproductive Damage in Male Rats: Modulation of Nrf2/HO-1 Signalling Pathway and Inhibition of Nf-KB-Mediated Testicular Apoptosis and Inflammation. Andrologia.

[74] Niazmand S., Mirzaei M., Hosseinian S., Khazdair M.R., Gowhari Shabgah A., Baghcheghi Y., Hedayati-Moghadam M. (2022). The Effect of *Cinnamomum cassia* Extract on Oxidative Stress in the Liver and Kidney of STZ-Induced Diabetic Rats. J. Complement. Integr. Med..

[75] Uslu G.A., Gelen V., Uslu H., Özen H. (2018). Effects of *Cinnamomum cassia* Extract on Oxidative Stress, Immunreactivity of INOS and Impaired Thoracic Aortic Reactivity Induced by Type II Diabetes in Rats. Braz. J. Pharm. Sci..

[76] Riós F., Quintero A., Piloni J., Cariño R., Reyes A. (2023). Compuestos Bioactivos de Canela y Su Efecto En La Disminución Del Síndrome Metabólico: Revisión Sistemática. Arch. Latinoam. Nutr..

[77] Zhang M., Chen Y., Yang M.-J., Fan X.-R., Xie H., Zhang L., Nie Y.-S., Yan M. (2019). Celastrol Attenuates Renal Injury in Diabetic Rats via MAPK/NF-ΚB Pathway. Phytother. Res..

[78] Wang R.-R., Chen X.-Y., Liao H.-L., Wan L., Li J.-M., Chen L.-L., Chen X.-F., Chen G.-R. (2010). The Relationship between the Expression of NF-KB, TGFbeta1, FN and Hepatic Fibrosis in Diabetic Rats. Zhonghua Gan Zang Bing Za Zhi.

[79] Reddy A.M., Seo J.H., Ryu S.Y., Kim Y.S., Kim Y.S., Min K.R., Kim Y. (2004). Cinnamaldehyde and 2-Methoxycinnamaldehyde as NF-ΚB Inhibitors from *Cinnamomum cassia*. Planta Med..

[80] Bagheri S., Sarabi M.M., Khosravi P., Khorramabadi R.M., Veiskarami S., Ahmadvand H., Keshvari M. (2019). Effects of Pistacia Atlantica on Oxidative Stress Markers and Antioxidant Enzymes Expression in Diabetic Rats. J. Am. Coll. Nutr..

[81] Bastos M.S., Del Vesco A.P., Santana T.P., Santos T.S., De Oliveira Junior G.M., Fernandes R.P.M., Barbosa L.T., Gasparino E. (2017). The Role of Cinnamon as a Modulator of the Expression of Genes Related to Antioxidant Activity and Lipid Metabolism of Laying Quails. PLoS ONE.

[82] Sharma S., Mishra A. (2017). Effects of Bark of Cinnamomum Zeylanicum on Hyperglycemia Induced Oxidative Stress and DNA Damage in Experimental Diabetic Rats. Int. J. Pharma Bio Sci..

[83] Attanayake A.P., Jayatilaka K.A.P.W., Mudduwa L.K.B., Pathirana C. (2019). β-Cell Regenerative Potential of Selected Herbal Extracts in Alloxan Induced Diabetic Rats. Curr. Drug Discov. Technol..

[84] Ruda-Kucerova J., Amchova P., Babinska Z., Dusek L., Micale V., Sulcova A. (2015). Sex Differences in the Reinstatement of Methamphetamine Seeking after Forced Abstinence in Sprague-Dawley Rats. Front. Psychiatry.

[85] Fedulova L.V., Basov A.A., Vasilevskaya E.R., Dzhimak S.S. (2019). Gender Difference Response of Male and Female Immunodeficiency Rats Treated with Tissue-Specific Biomolecules. Curr. Pharm. Biotechnol..

[86] Yamamoto T., Ohkuwa T., Itoh H., Sato Y., Naoi M. (2002). Effect of Gender Differences and Voluntary Exercise on Antioxidant Capacity in Rats. Comp. Biochem. Physiol. Part C Toxicol. Pharmacol..

[87] Kruger N.J. (1994). The Bradford Method for Protein Quantitation. Methods Mol. Biol..

[88] de Souza M.C., Vieira A.J., Beserra F.P., Pellizzon C.H., Nóbrega R.H., Rozza A.L. (2019). Gastroprotective Effect of Limonene in Rats: Influence on Oxidative Stress, Inflammation and Gene Expression. Phytomedicine.

